# Cyclic AMP-Induced p53 Destabilization is Independent of CREB in Pre-B Acute Lymphoblastic Leukemia Cells

**Published:** 2017-01-18

**Authors:** Rima Manafi Shabestari, Majid Safa, Mehdi Banan, Ahmad Kazemi

**Affiliations:** 1 *Department of Hematology, Faculty of Allied Medicine, Iran University of Medical Sciences, Tehran, Iran.*; 2 *Razi Drug Research Center, Iran University of Medical Sciences, Tehran, Iran.*; 3 *Genetics Research Center, University of Social Welfare and Rehabilitation Sciences, Tehran, Iran.*

**Keywords:** Apoptosis, cAMP, CREB, doxorubicin, p53

## Abstract

Elevated cAMP levels in B-cell precursor acute lymphoblastic leukemia (BCP-ALL) cells attenuate the doxorubicin-induced p53 accumulation and protect cells against apoptosis. cAMP responsive element binding protein (CREB) is a cAMP-stimulated transcription factor that regulates genes whose deregulated expression cooperate in oncogenesis. In the present study, we investigated the role of CREB on inhibitory effect of cAMP on apoptosis and p53 accumulation in BCP-ALL NALM-6 cells. To determine whether targeting CREB can modulate the effect of cAMP on doxorubicin-induced apoptosis, we knocked down CREB in NALM-6 cells using lentiviral CREB shRNA. Knocked down cells were treated with doxorubicin in the presence or absence of cAMP-elevating agents. p53 protein level and apoptosis were assessed by western blot analysis and flow cytometry, respectively. p53 protein expression was reduced in cells treated with combination of cAMP-elevating agents and doxorubicin in contrast to cells treated with doxorubicin alone even in CREB-knocked down cells. Apoptosis assay showed that the cAMP-elevating agents decreased doxorubicin-induced apoptosis in CREB-knocked down and control cells. Although, CREB plays a particularly important role in cAMP signaling pathway our data suggest that CREB does not mediate the inhibitory effect of cAMP on doxorubicin-induced apoptosis and p53 accumulation in BCP-ALL NALM-6 cells.

Acute lymphoblastic leukemia (ALL) is the most common childhood neoplasm and represents approximately 25% of cancers diagnosed in children (1). In spite of therapeutic improve-ments in ALL treatment strategies, about 20% of the patients show relapse which remains a major cause of morbidity and mortality in children. Previous studies showed that cAMP-responsive element binding protein (CREB) is overexpressed in majority of bone marrow samples from patients with ALL and AML (2). CREB is a transcription factor acts as a proto-oncogene in hematopoietic cells (3). Overexpression of CREB confers survival advantage to the cancer cells and associates with poor outcome in patients with AML (3). Moreover, suppression of CREB inhibits leukemic cell growth and induces apoptosis in some malignant conditions (4). In response to various signals such as growth factors, peptide hormones and neuronal activity CREB can be activated through phosphorylation at Ser133 by cAMP-dependent protein kinase A (5). cAMP is an important intracellular second messenger that mediates a wide variety of biologically distinct cellular processes including metabolism, cell differentiation, and apoptosis (6). It has been shown that cAMP signaling pathway can impede drug-induced apoptosis in leukemiccells (7). Likewise, downregulation of cAMP inhibits tumor growth and improve the effectiveness of anti-cancer drugs in treatment of glioblastoma and in killing glioblastoma stem cells (8). Previous studies have revealed that elevated levels of cAMP inhibit doxorubicin-induced apoptosis by dephosphor-ylation of p53 serine residues and attenuation of the p53 accumulation in BCP-ALL cells (9). In normal unstressed cells, p53 is present at low levels (10). In response to various cellular stresses such as DNA damage, oncogene overexpression, heat shock, and hypoxia, p53 becomes activated, that results in p53 protein level augmentation and transactivity (11). Activation of p53 determines the fate of the cell through various functions including cell cycle arrest, apoptosis, DNA repair and senescence (12). Anthracyclines such as doxorubicin that are used in treatment of ALL patients induce p53 accumulation in leukemic cells which can result in apoptosis (13). As CERB plays a critical role in mediating the cAMP effects, we hypothesized that cAMP exerts its inhibitory effect on p53 accumulation and apoptosis through CREB protein. To this end, CREB was knocked down using lentiviral CREB shRNAin BCP-ALL NALM-6 cells. 

## Material and methods

**Cell culture**

HEK 293T cells (kindly provided by Dr Frank Grosveld, Erasmus MC) used for the production of lentiviruses, were cultured in Dulbecco’s modified Eagle’s medium (DMEM), with 10% fetal bovine serum (FBS) and 1% penicillin-streptomycin. NALM-6 cells were cultured in RPMI-1640 supplemented with 10% FBS and penicillin/ streptomycin. Cells were maintained in an incubator at 37°C with 5% CO2 and 95% humidity (Memert, Germany). NALM-6 cells were divided into 3 groups including non-transduced cells, cells transduced with non-targeting scrambled shRNA-expressing lentiviruses and cells transduced with CREB shRNA-expressing lentiviruses. Each group of cells were treated as the following: no treatment (control cells), treatment with cAMP-elevating agents (forskolin 50 µM and IBMX 100 µM), treatment with doxorubicin alone (250 nM), and with doxorubicin in the presence of cAMP-elevating agents. In the latest group, forskolin and IBMX were added 30 min prior to doxorubicin addition. Forskolin is an activator of adenylyl cyclase and IBMX inhibits the phosphodiesterase-mediated degradation of cAMP to AMP, resulting in increased cAMP levels within the cells (14). 

**Lentivirus production and infection**

The day before transfection, 3 × 10^6^ HEK-293T cells were seeded in 10 cm dishes. The following day, cells were transfected with previously described CREB shRNA-expressing PLKO.1 lentiviralvectors (CREB shRNA-2 and shRNA-3) (15), scrambled shRNA-expressing control vector (SHC002V SIGMA) and GFP-expressing pLKO.1 vector along with packaging vectors (psPAX2 and pMD2.G) using FuGENE-6 transfection reagent (Promega, USA). Lentiviruses were harvested from supernatants at 48 and 72h after transfection and then filtered through 0.45μm PVDF filters. Produced lentiviruses were concentrated by ultracentrifugation (2 h at 100,000 g) in Beckman Optima L-90K ultracentrifuge (Beckman Coulter, USA). The virus-containing pellet was dissolved in DMEM, aliquoted and stored at -80°C. NALM-6 cells were transduced by adding 1 ml of concentrated virus supplemented with 2 μg/ml Polybrene to 4 × 10^5^ cells in 12-well plates. After 36 h the lentiviral supernatant was substituted by standard growth medium, and transduction efficiency was monitored by GFP expression at 48 h after removal of the virus containing medium.

**Sub-G1 DNA content analysis**

Late stage apoptotic or necrotic cells were detected by flow cytometry, using propidium iodide (PI) staining to determine the sub-G1 peak. Briefly, transduced and non-transduced NALM-6 cells (0.4 × 10^6 ^cells) were treated as mentioned above at 48 h after removal of the virus-containing medium. Cells were harvested 24 h after the treatment and washed once with PBS and fixed with 70% ethanol. Then cells were treated with 0.5 μg/ml RNase in PBS, and incubated for 5 min before staining with 50 μg/ml PI for 30 min. The cells were then analyzed using a BD FACSCalibur flow cytometer (16). Samples were gated for the singlet nuclei population by employing a FL2-A versus FL2-W dot plot and the gated events were then plotted as a FL-2A histogram. Sub-G1 DNA contents displa-ying hypo diploid nuclei were identified as apoptotic cells. The percentage of cells in the sub-G1 phase of the cell cycle was analyzed using BD Cell Quest software.

**Annexin-V apoptosis assay**

Annexin-V binding assay was done for flowcytometric detection of phosphatidyl serine expression on cells. Briefly, 48 h after removal of the virus-containing medium, transduced and non-transduced cells (0.4 × 10^6 ^cells) were treated as mentioned above. Cells were incubated for 24 h, then washed with PBS and resuspended in binding buffer and Annexin-V-Fluos (2 μl per sample). Cell suspension was incubated for 20 min in the dark and fluorescence was then measured using flow cytometry. Cell debris were excluded by setting a gate based on forward scatter versus side scatter plot, and annexin-V positivity was then assessed according to the gate. The data were expressed as percentage of positive cells for annexin-V (16).

**RNA extraction, and quantitative RT-PCR**

Total RNA was extracted from the shRNA-transduced and non-transduced cells using TriPure isolation reagent (Roche, Germany), according to the manufacturer’s instruction. 1μg of isolated RNA was used for preparation of cDNA using Revert Aid First Strand cDNA Synthesis kit (Thermo Scientific, Lithuania). The prepared cDNA was subjected to quantitative reverse-transcriptase polymerase chain reaction (qRT-PCR), using Maxima SYBR green master mix (Thermo Scientific, Lithuania) in the Rotor Gene 6000 Real Time PCR System (Corbett Research, Hilden, Germany). DNA was amplified during a 40-cycle PCR reaction with the following conditions: denaturation at 95°C for 15 s, annealing and elongation at 60°C for 60 s. The fold induction or repression was measured relative to the control and calculated after adjusting for reference gene *GAPDH* (16). Each sample was analyzed in triplicate. qRT-PCR was performed with CREB specific primers (forward primer: 5'-cacctgccatcaccactgtaa-3' and reverse primer: 5'- gctgcattggtcatggttaatgt-3').* GAPDH* was amplified using forward 5'-gaaggtgaaggtcggagtc-3' and reverse 5'-gaagatggtgatgggatttc-3' primers. 

**Western blot analysis**

CREB-knocked down and control cells were treated with doxorubicin in the presence or absence of cAMP elevating agents. Cells were centrifuged and cellular pellets were washed with cold PBS and lysed (5 × 10^6^ cells/aliquots) in 0.2 ml of RIPA buffer (10 mM Tris-HCl, pH 7.4, 150 mM NaCl, 5 mM EDTA, 1% Triton X-100, 0.1% sodium dodecyl sulfate, and 0.5% sodium deoxycholate) containing protease and phosphatase inhibitor cocktails (Sigma, Germany). After centrifugation at 13,000 g for 20 min at 4°C, the supernatant was collected. Protein concentrations were assessed by Bradford protein assay, and equal amounts of total cellular protein were subjected to 10% SDS-PAGE, according to the method of Laemmli. The gels were electro blotted onto nitrocellulose membranes (Hybond-ECL, Amersham Corp, UK). Membranes were then blocked with 5% nonfat dry milk in TBS containing 0.1% (v/v), Tween-20 for 1 h at room temperature, and incubated with specific primary antibodies overnight at 4°C (16). After 3 washes in TBS-Tween, membranes were probed with horse radish peroxidase-conjugated secondary antibodies. Then membranes were washed as above and proteins were visualized with ECL-chemilum-inescent kit (Amersham ECL Advance Kit, GE Healthcare, UK).

**Statistical analysis**

Data were analyzed using two-tailed student t-test. A p*-*value of <0.05 was considered to be statistically significant.

## Results

**Elevated cAMP levels attenuate the doxorubicin-induced apoptosis in NALM-6 cells**

**Fig. 1:**
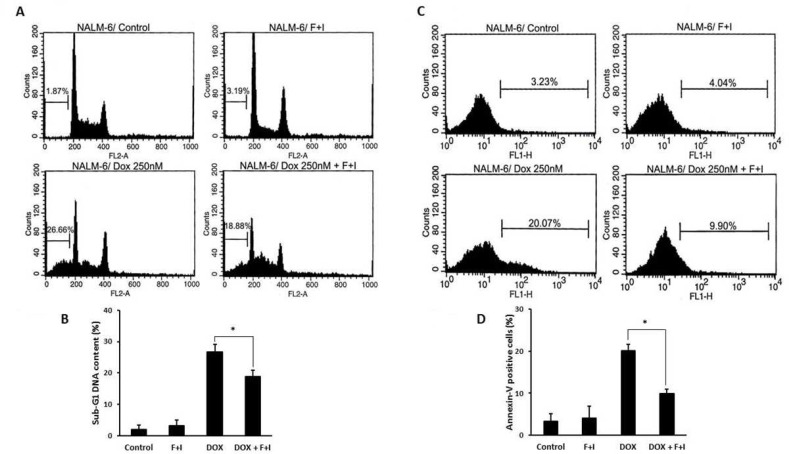
cAMP-increasing agents reduce doxorubicin-induced apoptosis in NALM-6 cells. A, B: elevation of cAMP levels resulted in decreased sub-G1 cell population. NALM-6 cells were pretreated with forskolin and IBMX for 30 min before the addition of doxorubicin. C, D: after 24 h cells were harvested and the percentage of cells in sub-G1 area was assessed by flow cytometry. (n = 3; * P=0.045, relative to cells treated with Dox alone).NALM-6 cells were treated with indicated agents for 24 h and apoptosis was determined using annexin V-FITC staining(n = 3; * P=0.04, relative to cells treated with Dox alon

In the present study, NALM-6 cells were treated with doxorubicin in the presence or absence of the cAMP-increasing agents, forskolin and IBMX. Then, sub-G1 DNA content analysis was performed using flow cytometry. As shown in Fig. 1A and 1B, cell population in sub-G1 phase was reduced in cells treated with doxorubicin in the presence of forskolin and IBMX compared with cells treated with doxorubicin alone. Moreover, the effect of cAMP-increasing agents on doxorubicin-induced apoptosis was further investigated by annexin-V staining assay. As shown in Fig.1C and 1D, treatment of cells with doxorubicin in combination with cAMP-elevating agents resulted in significant decrease in annexin-V positive cells compared with cells treated with doxorubicin alone. Taken together, these findings demonstrated that elevation of cAMP levels reduced the doxorubicin-induced apoptosis in NALM-6 cells.

**CREB knock down was not sufficient to prevent the inhibitory effect of cAMP on doxorubicin-mediated apoptosis**

**Fig. 2:**
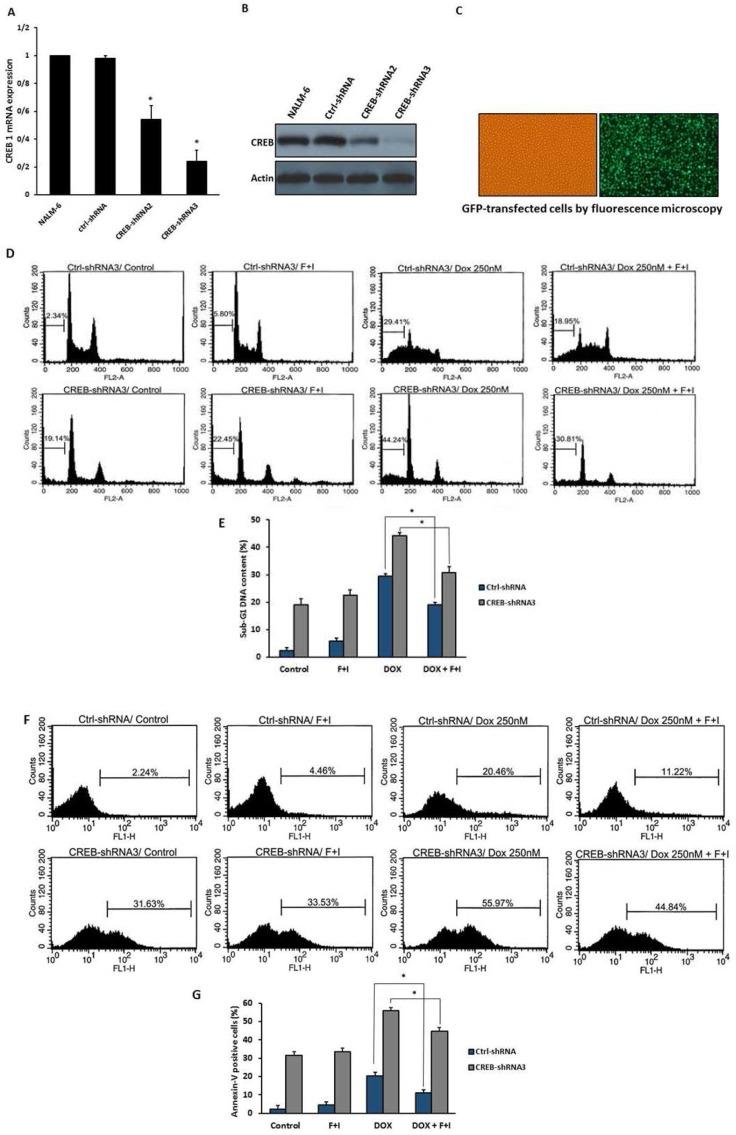
cAMP attenuates DNA damage-induced apoptosis in CREB-knocked down cells. A:NALM-6 cells were transduced with 2lentiviral vectors expressing CREB-targeting shRNA (CREB-shRNA2 and CREB-shRNA3) and scrambled non-targeting shRNA (Ctrl-shRNA). At 48 h after removal of the virus-containing medium, cells were harvested for total RNA extraction. CREB mRNA expression was measured using quantitative RT-PCR and normalized to the expression of *GAPDH* (n = 3; * P=0.014 for CREB-shRNA2 and 0.001 for CREB-shRNA3 relative to cells treated with Ctrl-shRNA). B:at 48 h after removal of the virus-containing supernatant cells were harvested for protein extraction. CREB prote in levels were assessed by Western blot analysis. Equal sample loading was verified by β-actin. C: at 48 h after replacement of lentiviral supernatant with standard growth medium, transduction efficiency was assessed by evaluation of GFP expression in NALM-6 cells. D, E:CREB-knocked down and control cells were treated with forskolin/IBMX 30 min prior to the addition of doxorubicin for 24 h. Cells were harvested and sub-G1 cell population was measured by flow cytometry(n = 3; *P=0.007 for Ctrl-shRNA+ (Dox+F+I) relative to cells treated with Ctrl-shRNA+Dox, P=0.019 for CREB-shRNA3+ (Dox+F+I) relative to cells treated with CREB-shRNA3+Dox). F, G:CREB-knocked down and control cells were treated with indicated agents for 24 h. Apoptotic cells were qualified using annexin-V and FACS analysis (n = 3; * P=0.046 for Ctrl-shRNA + (Dox+F+I) relative to cells treated with Ctrl-shRNA+Dox, P=0.022 for CREB-shRNA3+ (Dox+F+I) relative to cells treated with CREB-shRNA3+Dox

To ascertain the probable contribution of CREB in the suppressive effect of cAMP on doxorubicin-induced apoptosis, NALM-6 cells were transduced with lentiviruses expressing either the shRNA targeting CREB or scrambled control shRNA as described previously. As shown in Fig. 2A and 2B, CREB mRNA and protein levels were obviously reduced in cells transduced with CREB1 shRNA3 compared with cells transduced with CREB1 shRNA2. Transduction of GFP-lentivirus in NALM-6 cells revealed the high transduction efficiency as evaluated by fluorescence microscopy (Fig. 2C). Next, CREB-knocked down and control cells were treated with doxorubicin in the presence or absence of forskolin and IBMX. Cells were harvested after 24 h and cell death was assessed by PI staining and annexin-V binding assay using flow cytometry. As indicated in Fig. 2D and 2E, the percentage of cells in Sub G1 area was decreased in the presence of cAMP-increasing agents compared with cells treated with doxorubicin alone in both CREB-knocked down and control cells. In addition, the percentage of annexin-V positive cells was decreased in the presence of cAMP-increasing agents compared with cells treated with doxorubic in alone (Fig. 2F and 2G). Altogether, our data suggest that CREB knock down may not prevent the inhibitory effect of cAMP on doxorubicin-induced apoptosis**.**

**Inhibitory effect of cAMP on doxorubicin-induced p53 accumulation was not restored by CREB knock down**

**Fig 3:**
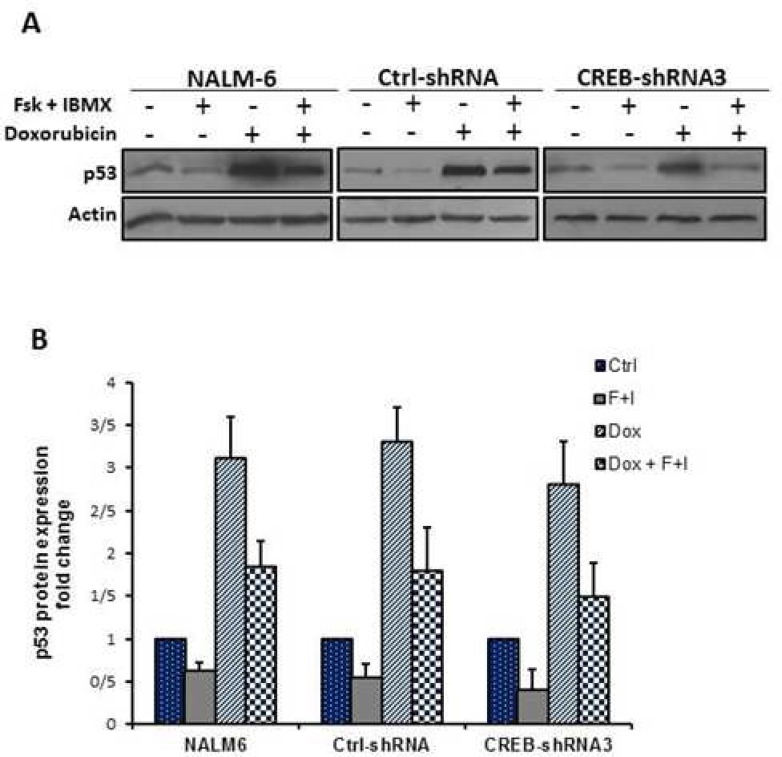
CREB knock down did not abrogate the inhibitory effect of cAMP on doxorubicin-induced p53 accumulation. A: CREB-knocked down and control cells were pretreated with or without forskolin/IBMX for 30 min before the addition of doxorubicin. After 4 h total cell lysates were prepared and western blot analysis was performed using antibodies specific to p53 and β-actin. B: the intensity of the bands was quantitated by densitometric analysis using Image J software and relative expression of p53 was calculated after normalizing to actin

It is known that elevation of intracellular cAMP levels can inhibit doxorubicin-induced p53 accumulation in NALM-6 cells. So, we wished to ascertain if CREB mediates the inhibitory effect of cAMP on p53 accumulation. To this end, 48 h after removal of the virus-containing medium, CREB-knocked down and control cells were treated with doxorubicin in the presence or absence of cAMP-increasing agents for 4 h. Subsequently, p53 protein levels were assessed by western blot analysis. As shown in Fig. 3, cAMP-increasing agents attenuate the doxorubicin-induced p53 accumulation even in CREB-knocked down cells.

## Discussion

Cyclic AMP signaling pathway is one of the most intensively studied areas in molecular biology. cAMP regulates many important cellular processes, and can also be implicated in intracellular signaling pathways. For instance, cAMP exerts its growth effects by interactions with the Ras-mediated MAP kinase pathways (17). The role of cAMP-increasing agents in modulation of drug-induced apoptosis has been investigated in different cell types with conflicting results. For example, increased cAMP levels induce apoptosis in B lymphocyte and granulose cells *(*18*, *19*). However, cAMP* *prevents chemotherapeutic drugs-induced apoptosis in leukemia cells, macrophages, MCF-7 breast cancer cells and pancreatic cells* (8, 20-23).Recent study by Gausdal et al. reported that cAMP-increasing agents protect acute promyelocytic leukemic blasts against anthracycline-induced apoptosis (24). In addition, it has been shown that cAMP exerts an inhibitory effect on arsenic-induced apoptosis in acute promyelocytic leukemia cells (25). We have already demonstrated that cAMP-elevating agents inhibit doxorubicin-induced p53 accumulation in ALL cells (9, 26). The tumor suppressor p53 mediates drug-induced apoptosis in response to DNA-damaging agents including doxorubicin. A previous study by Yang et al. reported that p53 status in breast cancer cells predicts cell survival or death following primary therapy (27). The critical role of p53 in suppression of malignant cells growth is reflected by the fact that downregulation of p53 leads to reduced apoptosis and enhanced tumor growth (28). Therefore, efforts to stabilize p53 in cancer cells led to develop novel strategies, including delivering wild type p53 to cancer cells using vectors (29), and inhibition of p53 degradation through impeding Mdm2-p53 interaction (30). Since cAMP has the ability to abrogate p53 accumulation and apoptosis in acute lymphoblastic leukemia cells (31), understanding the mechanism mediating this inhibitory effect is of great importance for effective therapeutic strategies in ALL. *Increased* *cAMP* *levels can activate* a number of various protein kinases including PKA that involves in different signaling pathways (32). Once activated by cAMP, the PKA translocates into the cell nucleus where it activates CREB. Previous studies indicated that CREB is a transcription factor implicated in abnormal survival and proliferation of leukemic blasts (3). Previous studies show that CREB protein is significantly overexpressed in the majority of the ALL patients at diagnosis (2). Thus, we hypothesized that CREB may be involved in mediating the inhibitory action of cAMP on apoptosis and p53 protein accumulation. As indicated in Fig.2D to 2G, the apoptosis was attenuated in cells treated with doxorubicin in the presence of cAMP-increasing agents compared with cells treated with doxorubicin alone in CREB-knocked down cells. In the present study, we found that CREB knock down did not alleviate the inhibitory effect of cAMP on doxorubicin-induced p53 accumulation, and therefore CREB is dispensable for cAMP-induced destabilization of p53 in BCP-ALL cells. cAMP-mediated effects may be transduced by exchange factor directly activated by cAMP (EPAC) that involves a variety of cellular functions such as cell adhesion, cell differentiation and proliferation, gene expression and apoptosis (17). Our previous study revealed that cAMP-induced p53 destabilization is independent of EPAC in pre-B acute lymphoblastic leukemia cells (26). However, in the recent study by Naderi et al. it was shown that PGE2-cAMP-PKA-axis could contribute to the protection of BCP-ALL cells from DNA damage-induced p53 accumulation and cell death (33).

Based on our findings, elevation of cAMP may act as a survival factor in BCP-ALL cells and further studies are required to precisely elucidate the molecular mechanisms involved in the cAMP–mediated inhibition of p53 accumulation and apoptosis. 
